# Ant Trail Pheromone Biosynthesis Is Triggered by a Neuropeptide Hormone

**DOI:** 10.1371/journal.pone.0050400

**Published:** 2012-11-30

**Authors:** Man-Yeon Choi, Robert K. Vander Meer

**Affiliations:** USDA-ARS, Center of Medical, Agricultural and Veterinary Entomology, Florida, United States of America; INRA-UPMC, France

## Abstract

Our understanding of insect chemical communication including pheromone identification, synthesis, and their role in behavior has advanced tremendously over the last half-century. However, endocrine regulation of pheromone biosynthesis has progressed slowly due to the complexity of direct and/or indirect hormonal activation of the biosynthetic cascades resulting in insect pheromones. Over 20 years ago, a neurohormone, pheromone biosynthesis activating neuropeptide (PBAN) was identified that stimulated sex pheromone biosynthesis in a lepidopteran moth. Since then, the physiological role, target site, and signal transduction of PBAN has become well understood for sex pheromone biosynthesis in moths. Despite that PBAN-like peptides (∼200) have been identified from various insect Orders, their role in pheromone regulation had not expanded to the other insect groups except for Lepidoptera. Here, we report that trail pheromone biosynthesis in the Dufour's gland (DG) of the fire ant, *Solenopsis invicta*, is regulated by PBAN. RNAi knock down of PBAN gene (in subesophageal ganglia) or PBAN receptor gene (in DG) expression inhibited trail pheromone biosynthesis. Reduced trail pheromone was documented analytically and through a behavioral bioassay. Extension of PBAN's role in pheromone biosynthesis to a new target insect, mode of action, and behavioral function will renew research efforts on the involvement of PBAN in pheromone biosynthesis in Insecta.

## Introduction

Pheromones are a subset of semiochemicals produced by organisms for communication within members of the same species. Since the first pheromone was identified over 50 years ago [1], pheromone research has been expanded tremendously in animal and plant taxa, especially insects [2], [3]. While pheromone identification and their elicited behaviors are well known for a wide variety of insects, the regulation of pheromone biosynthesis is poorly understood and is limited to some lepidopteran moths [4]–[6] For these moth species a neuropeptide hormone stimulates sex pheromone biosynthesis in female adults [7], [8]. This neuropeptide was named pheromone biosynthesis activating neuropeptide (PBAN), it is synthesized in the subesophageal ganglion (SG) and released into the hemolymph to reach a target site, e.g., a pheromone gland [8]–[10]. PBAN/pyrokinin genes appear to be ubiquitous in insects and produce three or four peptides in addition to PBAN [11], [12]. These peptides share a common functional epitope (FXPRL-NH_2_) or similar sequence at the C-termini [13], [14], which characterizes the PBAN/pyrokinin family of peptides [8], [15]. The PBAN/pyrokinin peptide family has been found in a variety of insect Orders, and to date over 200 PBAN/pyrokinin family peptides have been reported from over 40 species (from GenBank, unpublished). In addition to regulation of sex pheromone biosynthesis in female moths, several other physiological functions for this family of peptides have been demonstrated, for example: (a) induction of melanization in moth larvae [16], [17]; (b) induction of diapause egg in moths [18], [19]; (c) stimulation of visceral muscle contraction in cockroaches [20]; (d) acceleration of puparium formation in the flesh fly [21]; and (e) termination of development of pupal diapause in heliothine moths [22]. However, their involvement in the control of pheromone production has only been demonstrated for moth PBAN where it stimulates the biosynthesis of the sex pheromone [9], [10].

Like other social insects, the fire ant, *Solenopsis invicta*, evolved complex pheromone communication systems for resource procurement, maintenance of social structure, and territoriality. Much is known about their behavior and chemistry, but regulation of pheromone production has not been elucidated. This study aims to utilize molecular techniques, e.g., inhibition of gene expression – RNAi, as well as chemical analysis and bioassays to elucidate the involvement of PBAN on biosynthesis regulation of the of fire ant trail pheromone – essential for resource retrieval and colony emigration [23].

## Materials and Methods

### Fire ant colonies

All *S. invicta* samples were from monogyne (single functional queen) colonies collected in the Gainesville area (FL, USA) by nest excavation or by rearing colonies from newly mated queens. All colonies were maintained as described previously [24]. No specific permits were required for the described field collections and the collections did not affect endangered or protected species. The newly mated queens were collected in an area not protected in any way.

### SolinPBAN and the fire ant trail pheromone

**Figure 1:**
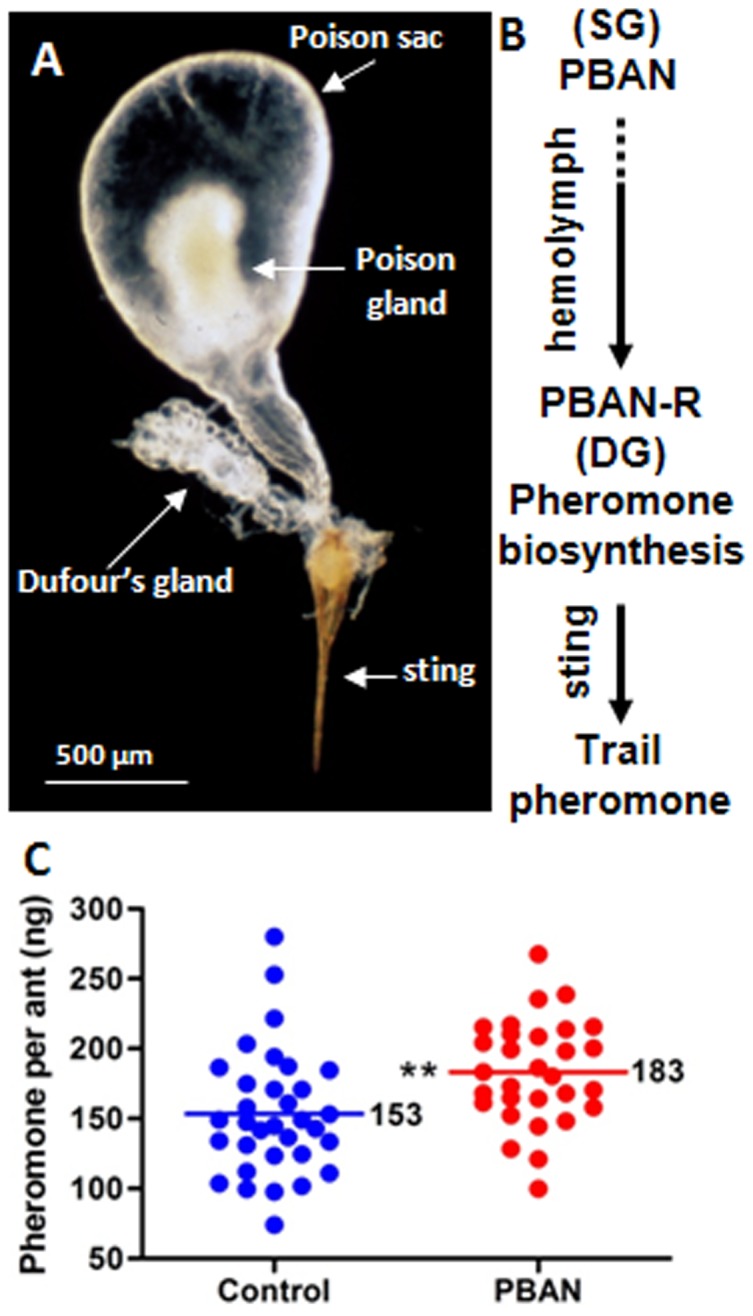
PBAN stimulates trail pheromone biosynthesis in the fire ant. (**A**) Photomicrograph of sting apparatus components -Dufour's gland (DG), poison gland/sac (PG), and sting in the worker. (**B**) Proposed pathway of trail pheromone biosynthesis in the worker. (**C**) SolinPBAN induces trail pheromone biosynthesis after SolinPBAN injection into workers. DGs were dissected from adult workers 6 h after SolinPBAN or saline (control) injection. The mean production of pheromone increased more than 30 ng (>19%) per ant after SolinPBAN injection. Data were analysed by two-tailed unpaired *t*-test (P = 0.0054, n≥35).

To test if *Solenopsis invicta* PBAN (SolinPBAN) stimulates trail pheromone biosynthesis, a saline control or SolinPBAN dissolved in saline was injected (10 pmol/50 nL/ant) into worker ants of approximately the same size (by inspection) and age (collected from the foraging area of rearing tray - age is related to task) using a Nanoliter 2000TM injector (World Precision Instruments) fitted with custom-pulled borosilicate needles. After injection, the fire ant workers were kept in a small plastic container with food and water until dissection and extraction of the trail pheromone for quantitation of the recruitment pheromone or for bioassay. A preliminary experiment indicated that the amount of trail pheromone 0, 1, 2, 4, 6 and 12 hours post PBAN injection was greatest after 6 hours (See Fig. S1B). This “incubation” time was chosen for the highly replicated (N≥35) saline vs. PBAN injection comparison (Fig. 1C).

### Gland dissection

All dissections were performed using a Zeiss Stemi SV 6 stereo microscope at 25× magnification. A drop of water was placed on the surface of a homemade silicon filled Petri dish (85 mm dia.). A decapitated ant was held at the anterior end of the gaster just below the petiole in a drop of water with #5 Dumont jewelers' forceps. With a second pair of forceps the last segment of cuticle on the dorsal side of the gaster was gently opened and removed, exposing the sting. The sting was grasped and gently pulled away, yielding the sting (S), the poison gland/sac (PG), and the Dufour's gland (DG). Forceps were cleaned in 10% sodium hypochlorite solution (bleach) between each dissection to avoid microbial and cross contamination. The DG is a small gland usually covered in fat bodies that is located at the base of the sting. The gland was pinched off at its base with the forceps, and checked to confirm its presence under microscope, then placed in an RNase free micro-centrifuge tube on Dry-Ice and kept at −80°C until RNA extraction. The Petri dish was cleaned with 10% bleach periodically between dissections. For GC analysis, a DG was placed in a 100 µL vial insert containing 50 µL hexane and 50 ng C16 as internal standard (IS). The insert was placed in a 2 ml vial containing a small amount of hexane, which acted to slow solvent evaporation. Samples were held at −20°C until analysis.

### Trail pheromone analysis

A GC (Agilent 6890N) equipped with a 30 m×0.25 mm i.d. DB-1 column, a flame ionization detector (FID) (Agilent), and autosampler (Agilent N10149) was temperature programmed 40°C to 285°C at 10°C/min with 5 min hold. Z,E-α-farnesene was identified based on prior GC-mass spectral and retention time analysis. Quantitation of Z,E-α-farnesene was achieved based on a peak area comparison with the IS (hexadecane). Due to different FID response factors for the sesquiterpene trail pheromone and the hydrocarbon IS, the results are relative to the IS – not absolute. The data were analyzed by non-parametric analysis as ranks (*t*-test, two-tailed) using GraphPad Prism 4.03 software.

### The fire ant PBAN receptor

Poly(A) RNA was isolated from whole bodies and heads of *S. invicta* workers and mature female alates for synthesis cDNA as described previously [25]. Synthesized cDNA was amplified with a degenerate primer set: 5′-ATGCAYCANGCNCANAAYTAYTAYYTNTT-3′ (MHTATNYYLF) and 5′-GCRTGRAANGGNGCCCARCADATRAARAA-3′ (FFICWAPFHA) mostly conserved in TM2 and TM7 domains from insect PBAN-Rs [25]–[27]. PCR was performed for 35 cycles at 95°C for 30 s, 50°C for 30 s, and 72°C for 1 min. The PCR product was directly sequenced by Interdisciplinary Center for Biotechnology Research (ICBR, University of Florida), and then the sequence result was used to design further gene-specific primers to find 5′- and 3′- ends of the fire ant PBAN-R cDNA. 5′-RACE (GeneRacer kit, Invitrogen) was performed using primers 5′-TGTGATGCCTCTTACTGCTGACGGTGGTCA-3′ (from nucleotide 1106) and 5′-TAGTAATTGGTGGCGGTGTGCATGGACTT-3′ (from nucleotide 588) by the 5′-RACE kit (Invitrogen). 3′-RACE was performed using primers 5′-TTYTTYATHTGYTGGGCNCC NTTYCA-3′ (from nucleotide 1199), 5′-CACCACGGTGAA TCCGCTTCTCTACAATA-3′ (from nucleotide 1336) and 5′-TCAAGTCGATGTTGCCTAAATATTGTATC-3′ (from nucleotide 1392). Then PCR products were inserted into a subcloning vector (TOPO-TA, Invitrogen) and confirmed the nucleotides by DNA sequence. Sequences of the receptor DNA and corresponding amino acids were analyzed by Genetyx software ver. 10 (Genetyx Corporation) and PredictProtein software [28]. The ORF of PBAN-R cDNA was amplified using the sense primer of 5′- GTCGCGGCCGCTAAAGCAGAATGTTTTCG AGTAATACG-3′ (from nucleotide 255 including the *Not*I site) and 5′-AGGCTCTAGATTGTCTTAATTCGA GAGGCGACGTTCT-3′ (from nucleotide 2122 including the *Xba*I site). The PCR product was ligated into a pIBV5His vector (Invitrogen) for expression in Sf9 cells as described previously [25].

### SolinPBAN-R/peptide binding assays

All peptides used in this study were synthesized or purchased from Sigma Genosys or Peninsula Laboratories. The preparation of cells, peptides, and Fluo-4AM with hymenopteran saline [29] followed a previously described method [25]. Cell fluorescence intensity in a 96-well cell-culture was measured using a plate reader (BMG's NovoStar) equipped with filters (excitation: 485 nm and emission: 520 nm), and one pipetter and two injectors. Fluorescence measurements from each well were taken every 10s. After 30s PBAN or FXPRL peptide (10 µL) was added by pipetter and fluorescent changes were measured for up to 3 min. Then, 1 µM ionomycin (5 µL) was added into the cells to obtain a maximum fluorescence reading. The effect of ligand-exposure was expressed relative to the maximum value obtained with ionomycin. Data were analyzed using Microsoft EXCEL as described previously [25] and measured EC_50_ values of ligands were determined using GraphPad Prism 4.03 software.

### RNAi suppression of SolinPBAN and SolinPBAN-R gene expression

Fire ant PBAN ( = SolinPBAN) dsRNA was constructed with 5′-T7-appended PCR primers (5′-TAATACGACTCACTATAGGGACCGTCGACAACCGACTTAC-3′and 5′-TAATACGACTC ACTATAGGGGACTCTCAAGAGGTGGTGGC-3′) to amplify a 506-bp PBAN DNA fragment, which serves as the template for dsRNA synthesis using the MEGAscript RNA kit (Ambion). Fire ant PBAN-R (SolinPBAN-R) dsRNA was constructed by specific primers (5′-TAATACGACTCACTATAGGGACCGTCGACAACCGACTTAC-3′ and 5′-TAATACGACTCACTATAGGGGACTCTCAAGAG GTGGTGGC-3′) to amplify a 510-bp DNA template for PBAN-R dsRNA synthesis. GFP dsRNA was constructed by specific primers (5′-TAATACGACTCACTATAGGGACGTAAACGGCCACAAGTTC-3′ and 5′-TAATACGA CTCACTATAGGGTGCTCAGGTAGTGGTTGTCG-3′) to amplify a 546-bp DNA template for GFP dsRNA using the same kit as above. The purified dsRNAs were dissolved in nuclease-free water and injected into workers using the same injector as above. Adult workers were injected with 1 µg (50 nL) of SolinPBAN or SolinPBAN-R dsRNA and incubated for 24, 48, and 72 h. After injection, fire ants were kept as described above until Br-SG or DG dissection. Br-SGs were dissected to isolate total RNA for SolinPBAN transcription by RT-PCR. Dissected DGs were used for trail pheromone analysis and for isolation of total RNA and determination of SolinPBAN-R transcription levels by RT-PCR as described below.

### RT-PCR for SolinPBAN and SolinPBAN-R

**Figure 2:**
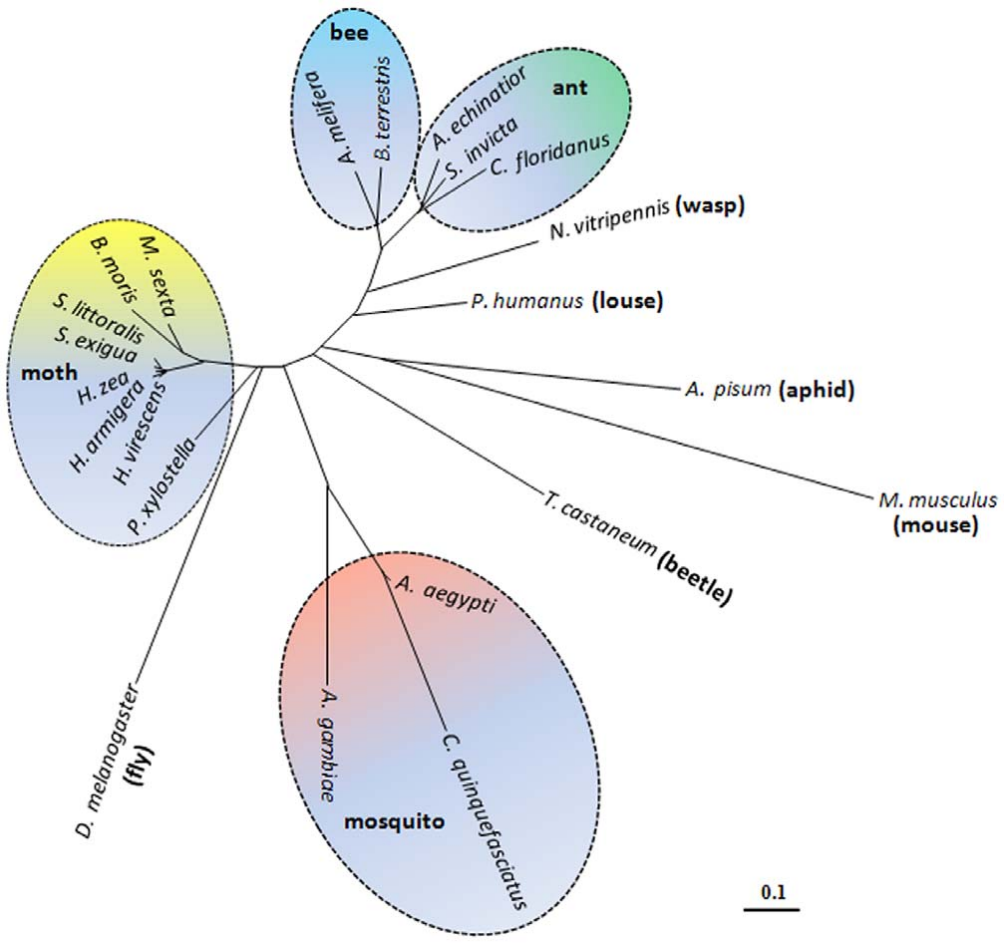
Phylogenetic tree of receptors for PBAN, PK2 and neuromedin U (NmU). The tree was made by Genetyx-Tree software (ver. 10) with the neighbor-joining distance phylogram method using bootstrap 1,000 replicates. Accession number of species: *Acromyrmex echinatior* (EGI70561), *Acyrthosiphon pisum* (XP_001950091), *Aedes agypti* (XP_001657210), *Anopheles gambiae* (AAX84798), *Apis melifera* (NP_001091688), *Bombus terrestris* (XP_003395149), *Bombyx mori* (NP_001036977), *Camponotus floridanus* (EFN62034), *Culex quinquefasciatus* (XP_001861460), *Drosophila melanogaster* (CG8795), *Helicoverpa armigera* (AAW47417), *Helicoverpa zea* (AAP93921), *Heliothis virescens* (ABU93812), *Manduca sexta* (ACQ90219), *Mus musculus* (NP_034471), *Nasonia vitripennis* (XP_001600587), *Pediculus humanus* (XP_002424393), *Plutella xylostella* (AAY34744), *Solenopsis invicta* (JX657040), *Spodoptera exigua* (ABY62317), *Spodoptera littoralis* (ABD52277), *Tribolium castaneum* (EEZ97728).

Total RNA was isolated after DNase treatment from the following fire ant tissues: head, Br-SG and abdomen, the PDS (PG, DG, and sting together), PG, DG, sting, and ventral nerve cord (VNC) using the PureLinkTM RNA Kit (Invitrogen) (Fig. 2C). Generally, each total RNA was used to synthesize cDNA using 3′-RACE kit or an antisense primer (5′-TGTAACGCGCC AATTCCGATCCC GTGAAT-3′) by SuperScript RT® III (Invitrogen). The 1st cDNA was synthesized from 5 µg total RNA from the head, Br-SG, and adult body minus head, 1 µL (1/20) was used for PCR amplification. The 1st cDNA was synthesized from total RNA (∼1200 ng) from PDS, DG, sting, and VNC, and 1 µL (1/20) was used for PCR amplification. Each cDNA was used to amplify a 313-bp DNA fragment of SolinPBAN-R with a specific primer set (5′-CACCACGGTGAATCCGCTTCTCTACAATA-3 and 5′-TGTAACGCGCCAATTCCGA TCCCGTGAAT-3′). A 100-bp fragment of the fire ant 18S rRNA was also amplified for a positive control as described previously [30]. PCR was performed as follows: 35 cycles at 95°C for 30s, 50°C for 30s, and 72°C for 1 min.

RT-PCR for SolinPBAN expression was performed with total RNA isolated from Br-SGs of workers (>15 ants/treatment) after they were injected (1 µg/50 nL/ant) with PBAN dsRNA, nuclease-free water, or in a separate experiment dsGFP and incubated for 24 h, 48 h and 72 h. The total RNA (20 ng) was used to amplify a 501-bp DNA fragment of PBAN with a PBAN specific primer set (5′-AGGAATTCCGTTAATCGTGC-3′ and 5′-GTTGCGTAATTGACGTCCG-3′). A 100-bp fragment of the fire ant 18S rRNA gene was also amplified as a positive control using primers (5′-CCCGTAATCGGAATGAGTACACTTT-3′ and 5′-ACGCTATTGGAGCTGGAATTACC-3′). The one-step RT-PCR was performed as follows: 1 cycle at 50°C for 30 min, 40 cycles at 94°C for 30 s, 53°C for 30 s, and 72°C for 1 min, then 72°C for 5 min using RT/Taq mix polymerase (Invitrogen). Then the second PCR was conducted with 1 µL of the above PCR products and the same primers using the following cycles: 1 cycle at 95°C for 3 min, 33 cycles at 95°C for 30 s, 60°C for 30 s, and 72°C for 1 min, then 72°C for 5 min using iTaq DNA polymerase (Bio-Rad). Then, part of the PCR products were checked for the amplification using 1.5% agarose gel electrophoresis and visualized using ethidium bromide under a UV light. The expected PCR products were purified and cloned, then confirmed by DNA sequence at ICBR.

RT-PCR for SolinPBAN-R expression was performed with total RNA isolated from DGs of workers (>15 ants/treatment) after workers were injected (1 µg/50 nL/ant) with SolinPBAN-R dsRNA, nuclease-free water, or in a separate experiment dsGFP at 24 h, 48 h and 72 h. The total RNA (20 ng) from each treatment was used to synthesize 1^st^ strand cDNA with the PBAN-R specific anti-sense primer (5′-TGTAACGCGCCAATTCCGATCCCGTGAAT-3′) and 18S (positive control) anti-sense primer (5′-ACGCTATTGGAGCTGGAATTACC-3′) using SuperScript RT® III (Invitrogen) following the manufacturer's procedure. Then, PCR amplification was performed with 2 µL of the 1^st^ strand cDNA above as a template to amplify a 313-bp DNA fragment of PBAN-R with the specific primer set (5′-CACCACGGTGAATCCGCTTCTCTACAATA-3 and 5′-TGTAACGCGCCAATTCCGATCCCGTGAAT-3′). A 100-bp fragment of the fire ant 18S rRNA was also amplified as a positive control as previously described. The PCR condition was 95°C for 3 min, 33 cycles at 95°C for 30 s, 50°C for 30 s, and 72°C for 1 min, then 72°C for 5 min using iTaq DNA polymerase (Bio-Rad). The expected PCR products were purified and cloned, then confirmed by their DNA sequence at ICBR.

### Reduced trail pheromone production and the orientation bioassay

A queen right colony collected and maintained in the laboratory as described above for approximately 4 weeks was selected for the bioassay. The trail orientation bioassay was modified from a previously described assay [31], [32]. A colony cell (14 cm Petri dish with a central hole in the lid and a layer of moistened Castone© in the bottom) containing the queen, brood and workers was transferred to a clean rearing tray (50×37×12 cm deep) with a water tube and starved for approximately 24 h. A tongue depressor (12 cm) was used as a ramp to lead worker ants from the tray floor to a plexiglass platform (8×11×8 cm tall; Fig. S4), then across another tongue depressor (7 cm) to a second platform (same size as the first) holding food (a dead cricket and 10% sugar water in a cotton ball). A piece of paper (size of platform) was placed on the first platform and the ants were then allowed to form a natural trail from their colony cell to the food platform. Bioassays were conducted by replacing the original paper with a test sheet (size of platform). Test sheets had two curved pencil lines (ca. 10 cm) drawn such that they ended at the mid-point of the short sides of the paper. The test papers were prepared for a bioassay by streaking one pencil arc with 10 µL hexane (10 µL Hamilton© syringe) and the other arc streaked with a DG extract (concentration expressed as DG equivalents). A bioassay was scored positive if a least one foraging ant followed the treatment arc to the food source within 1 min. The test paper was then removed and the original paper was returned to the platform allowing the ants to return to their natural trail between bioassays.

## Results and Discussion

The DG is the source of the fire ant trail pheromone [33], which is released through the sting (Fig. 1A). The most abundant volatile trail pheromone component is Z,E-α-farnesene (Fig. S1A) [34]. This component is responsible for the orientation of the ants along a pheromone trail. Our earlier studies on the trail pheromone [34] coupled with our recent characterization of the PBAN/pyrokinin family of peptides from fire ants [11], [29], [30], [35], provided the foundation for the discovery that *Solenopsis invicta* PBAN (SolinPBAN) regulates trail pheromone biosynthesis in the DG (Fig. 1B).

### SolinPBAN and the fire ant trail pheromone

Many moth sex pheromones are biosynthesized in epithelial glands located in the last abdominal segments, usually with no lumen [5], [36]. This type of gland/pheromone system facilitated bioassays needed to determine the effect of PBAN by measurement of pheromone present or absence in decapitated or neck-ligated female moths [7], [8]. However, exocrine glands with a lumen, e.g. DG [37], maintain pheromone levels within a range through biosynthetic activation and deactivation. This variability presents challenges to measure differences from experimental hormone stimulation. To determine if SolinPBAN stimulates trail pheromone production in the DG we measured Z,E-α-farnesene levels in adult worker DGs after SolinPBAN or saline (control) injections. To maximize potential treatment and control differences we first determined that 6 h was an optimal post injection incubation period (Fig. S1B). A large-scale experiment using this incubation time then showed that SolinPBAN injected worker ants produced significantly greater amounts of trail pheromone than ants injected with saline (Fig. 1C). About 85% of SolinPBAN treatments had trail pheromone levels above the normal range, as defined by the mean saline control, and presumably closer to the DG capacity limit (Fig. 1C). This result strongly supports SolinPBAN stimulation of trail pheromone biosynthesis in the DG, and suggests that a receptor for SolinPBAN could be located in the DG as a potential SolinPBAN target site.

### The fire ant PBAN receptor

Insect PBAN receptors are classified in the G-protein-coupled receptor (GPCR) superfamily and many have been identified by sequence homology [12], [26], [38], [39] and/or by functional expression in insects [25], [27], [40]–[47]. To investigate the potential SolinPBAN target site(s) in fire ants and neuropeptide binding preferences, we identified the SolinPBAN receptor (SolinPBAN-R) using degenerate primers (deposited the nucleotide and amino acid sequences in GenBank with accession number, JX657040). As expected SolinPBAN-R is closest to other ant PBAN receptors, followed by bees, and then moths (Fig. 2), but SolinPBAN-R transmembrane (TM) domains are highly conserved with those from different insect Orders (Fig. S2).

### SolinPBAN-R/peptide binding assays

**Figure 3:**
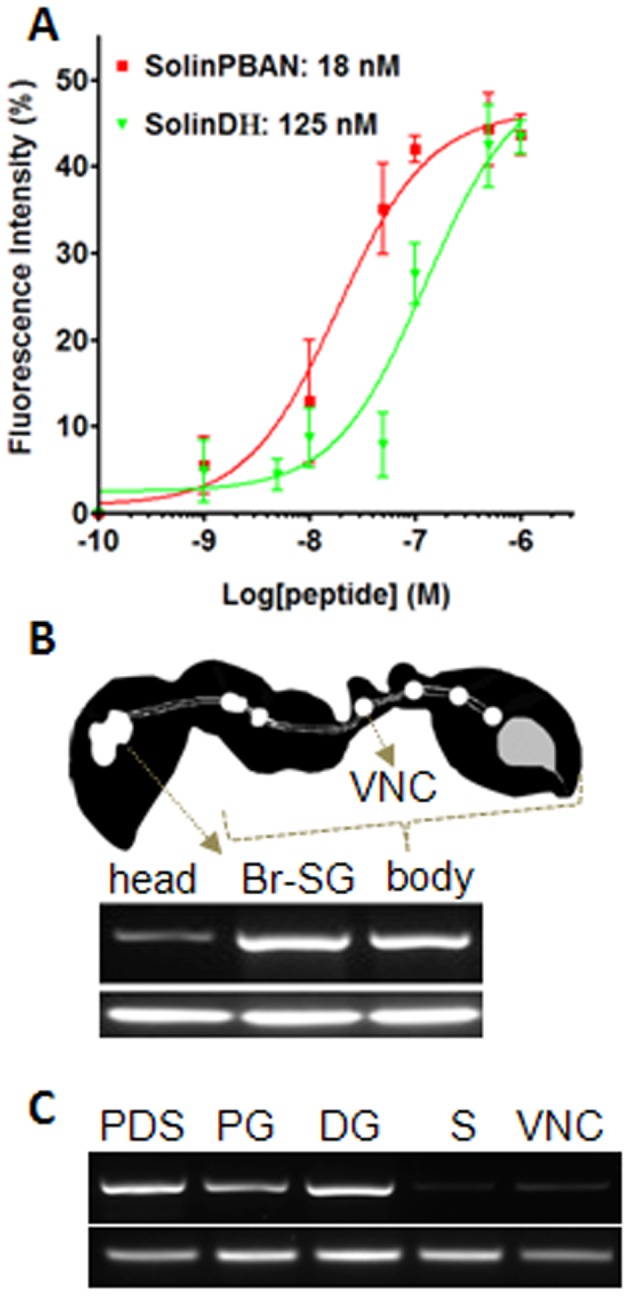
SolinPBAN-R binding specificity and expression. (**A**) Half-maximal effective concentration (EC_50_) values of synthetic peptides, SolinPBAN and SolinDH, of the fire ant to bind with SolinPBAN-R. The y-axis indicates the peak peptide fluorescence value relative to ionomycin treatment (mean ± s.e.m; N≥4). (**B**) Photomicrograph of worker ant and SolinPBAN-R gene expression from head, Br-SG and body (without head). (**C**) SolinPBAN-R gene expression from the complex of PG, DG and sting (PDS), PG, DG, sting (S) and ventral nerve cord (VNC). Fire ant 18S rRNA was used for the positive control.

The PBAN/pyrokinin family of peptides and their receptors are currently classified as PK1 or PK2. Diapause Hormone (DH) peptides and their receptors are classified as type PK1. PBAN and other PBAN family peptides, and their receptors are classified as type PK2 [12]. To verify our SolinPBAN-R assignment we determined binding affinities for a number of PK1 ( = DH type) and PK2 ( = PBAN type) neuropeptides to SolinPBAN-R (Table 1). We expressed SolinPBAN-R in *Spodoptera frugiperda* 9 (Sf9) insect cells [25], which enabled binding specificity studies by measurement of calcium-based fluorescence intensity (Fig. 3A and Table 1). The two types of peptides can be distinguished by their different binding affinities (half-maximal effective concentration, EC_50_) to SolinPBAN-R. SolinDH was 7 times less active than SolinPBAN in binding with SolinPBAN-R (Fig. 3A) and the EC_50_ values of other PK1 peptides from *Drosophila* and moth were >50 time less active than SolinPBAN (Table 1), supporting preferred SolinDH binding to a PK1 receptor [12], [48], [49]. On the other hand, the low EC_50_ binding values of SolinPBAN and the moth PBAN, HelzePBAN (*Helicoverpa zea* PBAN), to SolinPBAN-R support its designation as a PK2 type receptor (Table 1). The EC_50_ value of SolinPBAN was 3 times less than HelzePBAN (18 nM vs. 56 nM, respectively), indicative of at least family ligand/receptor fidelity. The strong binding affinity of SolinPBAN confirmed SolinPBAN-R as the receptor for SolinPBAN in the fire ant. The SolinPBAN/pyrokinin gene is expressed in all life stages in fire ant, from egg to adult [50]; however, the disposition of the four SolinPBAN/pyrokinin peptides in each life stage, as well as the possibility of additional receptors is unknown and currently under investigation.

**Table 1:** Binding activity of PBAN/pyrokinin and related peptides to SolinPBAN-R.

Species	Peptide*	Amino acid sequence**	EC_50_ ***
*S. invicta*	SolinPBAN	GSGEDLSYGDAYEVDEDDHPLFVPRL	18 nM
	SolinDH	TSQDIASGMWFGPRL	125 nM
*H. zea*	HelzePBAN	LSDDMPATPADQEMYRQDPEQIDSRTKYFSPRL	56 nM
	HelzePGN24 (DH)	NDVKDGAASGAHSDRLGLWFGPRL	>1 µM
*D. melanogaster*	DromePK2	SVPFKPRL	163 nM
	DromePK1	TGPSASSGLWFGPRL	>1 µM
*L. maderae*	LPK	PETSFTPRL	36 nM

* Peptides: PBAN (pheromone biosynthesis activating neuropeptide), DH (diapause hormone), PGN (PBAN-encoding gene neuropeptides), PK (pyrokinin), LPK (leucopyrokinin).

** All peptides are amidated in the C-termini.

*** EC50: half-maximal effective concentration.

We localized SolinPBAN-R in the ant by measuring receptor gene transcription levels in various tissues (Fig. 3B). Strong SolinPBAN-R expression was detected in the Br-SG and body (Fig. 3B). Most interesting was the result obtained from the body, because the DG resides in the abdominal part of the body of the fire ant (Fig. 1A). Further investigation of the abdomen showed SolinPBAN-R expression predominantly in the DG and moderately in the poison gland/sac (PG) (Fig. 3C). Strong PBAN-R expression in the DG supports it as a target site for SolinPBAN in the fire ant. Expression of SolinPBAN-R in the Br-SG (Fig. 3B) and moderate expression in the PG (Fig. 3C) suggests other physiological roles for the SolinPBAN family of peptides. In an earlier study the expression of SolinPBAN mRNA was detected in all developmental stages, from embryo to adults [50], indicating probable stage-specific functions in the fire ant.

### RNAi suppression of SolinPBAN and SolinPBAN-R gene expression

**Figure 4:**
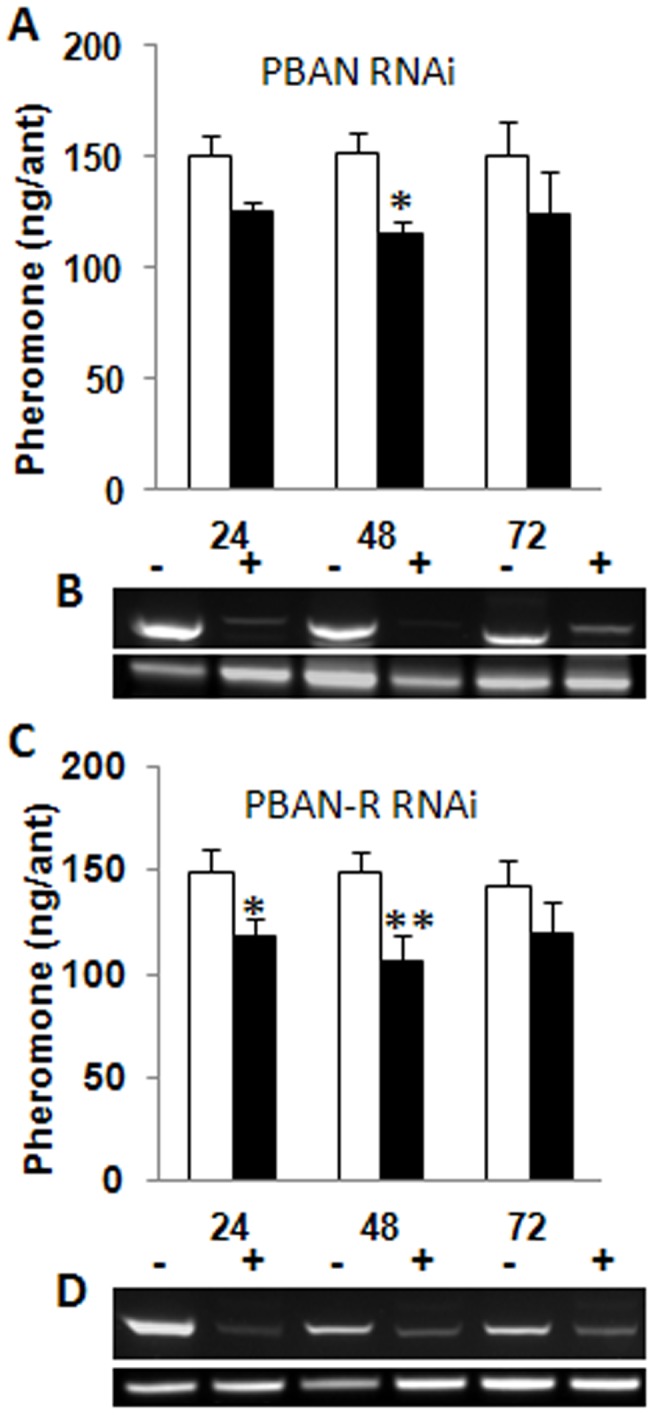
RNAi effects on trail pheromone production and SolinPBAN and SolinPBAN-R gene expressions in workers. (**A**) Suppression of trail pheromone production and (**B**) SolinPBAN gene expression from Br-SG of workers at 24, 48 and 72 h post-injection of nuclease-free water (−) or SolinPBAN dsRNA (+). (**C**) Suppression of trail pheromone production and (**D**) SolinPBAN-R gene expression from DG of workers at 24, 48 and 72 h post-injection of nuclease-free water (−) or SolinPBAN-R dsRNA (+). Data were analyzed by two-tailed unpaired *t*-test (mean ± s.e.m., asterisks indicate, SolinPBAN RNAi at 48-h, P = 0.0479, n≥10, and SolinPBAN-R RNAi at 24-h, P = 0.0302, n≥10, at 48-h, P = 0.0136, n≥10, respectively). Fire ant 18S rRNA was sued for the positive control.

To further test the hypothesis that SolinPBAN is involved in fire ant trail pheromone biosynthesis, we used RNA interference (RNAi) to knock down SolinPBAN and SolinPBAN-R in adult workers (Fig. 4). Worker ants injected with SolinPBAN dsRNA were incubated for 24, 48, and 72 h prior to DG dissection for trail pheromone analysis. The SolinPBAN RNAi significantly inhibited trail pheromone production at 48 h, but results at 24 and 72 h post-injection were not significantly different from controls (Fig. 4A). SolinPBAN gene transcription levels were determined from dissected Br-SGs from worker ants at 24, 48, and 72 h post-injection of SolinPBAN dsRNA. SolinPBAN expression was inhibited at all three time periods (Fig. 4B), with maximum effect at 48 h, which corresponded to the time of greatest pheromone reduction (Fig. 3A). SolinPBAN transcription was clearly inhibited after 24 h post-injection; however it is possible that residual SolinPBAN and/or SolinNPs synthesized in the SG prior to RNAi injection stimulated some pheromone production in the DG during the first 24 h. Previous research using moth PBAN RNAi gave a similar result for reduction of sex pheromone production in *H. zea* [50]. Injection of the non-specific dsRNA control, GFP dsRNA, did not reduce transcription of SolinPBAN (Fig. S3).

The effect of SolinPBAN-R RNAi on trail pheromone production was measured 24, 48, and 72 h after workers were injected with SolinPBAN-R dsRNA or a nuclease-free water control. Pheromone production was significantly reduced at 24 and 48 h sampling times (Fig. 4C). At 72 h post injection, pheromone production was decreased but not significantly. To determine if SolinPBAN-R RNAi was affecting SolinPBAN-R transcription in DGs, we dissected DGs from worker ants injected with SolinPBAN-R dsRNA after 24, 48, and 72 h post-injection and observed SolinPBAN-R expression levels (Fig. 4D). SolinPBAN-R expression in DGs was reduced for all time periods, especially evident at 24 h and 48 h post-injection (Fig. 4D), corresponding to when the greatest pheromone reduction occurred (Fig. 4C). Injection of the non-specific dsRNA control, GFP dsRNA, did not reduce transcription of SolinPBAN-R (Fig. S3) and gave results indistinguishable from those of the nuclease-free water.

The RNAi impact of SolinPBAN and SolinPBAN-R at 48 hr post-injection resulted in significant reduction in pheromone production, which was correlated with reduced transcription levels of the target genes and decreased number of SolinPBAN receptors, respectively. PBAN-R RNAi has already been shown to inhibit PBAN-R transcription in moths [47], [51], [52], resulting in reduced sex pheromone production, which is similar to our results for SolinPBAN-R RNAi and reduced trail pheromone production in fire ants.

### Reduced trail pheromone production and the orientation bioassay

Finally, we used a trail orientation behavioral assay to evaluate if the level of decreased trail pheromone resulting from RNAi suppression of SolinPBAN-R and resulting reduced levels of Z,E-α-farnesene could be detected behaviorally. As described earlier, workers were injected with SolinPBAN-R dsRNA then after 48 h their DGs were dissected. Serial dilutions of DG extracts (treatment and control) and orientation bioassays with these extracts were used to determine the minimum detectable DG worker equivalent for treatment and control samples (Fig. S4). Workers could not detect DG extracts at 0.001 DG equivalents of treatments, whereas workers readily followed the same DG equivalent concentration of the control (Movie S1 and S2). These results demonstrate a behavioral consequence associated with the negative effect of PBAN-R RNAi on the regulation of trail pheromone biosynthesis (Fig. 4C).

## Conclusions

Taken together, our results demonstrate that the neuropeptide, SolinPBAN, stimulates trail pheromone biosynthesis in fire ants. The consequences of this discovery are: 1) after >20 years, the scope of PBAN's activity is broadened to insect groups other than lepidopteran species; 2) the behavioral type of pheromone acted on is extended from only sex pheromones to include ant trail pheromones; 3) biosynthetic regulation is extended from fatty acid to the highly versatile isoprenoid pathway; and 4) molecular level manipulation of RNAi of PBAN and PBAN-R suggests the possibility of novel control methods for insect pests (47). We anticipate that the research model presented here will lead to yet broader elucidation of PBAN's role in insect pheromone biosynthesis, as well as additional function(s).

## Supporting Information

Figure S1 **SolinPBAN stimulates production of trail pheromone in the fire ant.** Forager (oldest) workers were used in this experiment. (**A**) Gas Chromatogram of Dufour's Gland (DG) extract showing Z,E-α-farnesene (ZEF, trail pheromone), two homofarnesenes and a C16:0 internal standard. Approximately 160 ng ZEF was detected in each DG. (**B**) The optimal incubation time after SolinPBAN injection for recruitment pheromone production was determined by calculating the percent change in pheromone concentration for treatments versus saline controls at six incubation times. Means (± s.e.m., P = 0.0158, n≥10) are shown. A six h incubation period after SolinPBAN injection showed a significant increase in ZEF.
(PPTX)

Figure S2 **Alignment of PBAN and NmU receptors.** Ant (*S. invicta*), bee (*A. mellifera*, NP_001091688), fly (*D. melanogaster*, CG8795), mosquito (*A. gambiae*, AAX84798), moth (*H. zea*, AAP93921), beetle (*T. castaneum*, EEZ97728), louse (*P. humanus*, XP_002424393), aphid (*A. Pisum*, XP_001950091) and mouse (*M. musculus*, NP_034471). The alignment was made by Genetyx-Tree software (ver. 10). The transmembrane domains for the *S. invicta* PBAN-R are indicated by double dashes above the aligned sequences. Dashed lines indicate spaces needed to optimize alignment.
(PPTX)

Figure S3 **GFP RNAi effects on SolinPBAN and SolinPBAN-R gene expression in workers.** Gene transcription levels of SolinPBAN expression in the Br-SG (upper) and SolinPBAN-R expression in DGs (middle) of workers at 24, 48 and 72 h post-injection of GFP dsRNA. Expression of the fire ant 18S rRNA, the positive control, is shown in the bottom row (see Materials and Methods for details).
(PPTX)

Figure S4 **Photo of orientation bioassay.** Ants are forced to develop a pheromone trail up the ramp and across the platform to the food reward. Bioassays are performed by replacing the paper on the platform with another paper streaked with control and treatment trails, then observing the behavior of the previously trailing ants (see Materials and Methods for details).
(PPTX)

Movie S1 **Positive ( = control) trailing bioassay.** A hexane extract of Dufour's glands (DG) from saline injected workers was applied to the trailing bioassay curve marked ‘T’ (10 µL of a 0.001 DG equivalent extract). Positive trailing behavior is indicated here by the ants readily following the experimental Control trail.
(WMV)

Movie S2 **RNAi treatment ( = SolinPBAN-R (SolinPR) dsRNA) trailing bioassay.** A hexane extract of Dufour's glands (DG) from dsPBAN-R injected workers was applied to the trailing bioassay curve marked ‘T’ (10 µL of a 0.001 DG equivalent extract). Negative trailing behavior is indicated here by the ants not detecting the experimental Treatment trail.
(WMV)
